# Cholelithiasis1Read at the meeting of the Roswell Park Medical Club, February 1, 1904.

**Published:** 1904-04

**Authors:** W. E. Dignen

**Affiliations:** Buffalo, N.Y.


### Cholelithiasis.[1]

By W. E. DIGNEN, M. D., Buffalo, N. Y.

### HISTORY.

GALLSTONES are seen so frequently nowadays, that it is astonishing to find so little mention of this condition among medical authors of antiquity, who are otherwise renowned for the accuracy of their observations. No data are given in the writings of antiquity that allow us to infer with certainty that the physicians of that day knew of this disease, nor is there any description that would lead us to believe that cases described were sufferers from gallstones. In the writings of Hippocrates and Galen, it is true, we find a few brief communications on pain in the hepatic region, on icterus as a result of constipation without fever, but these descriptions, as compared to the precise and exact communications on renal colic, convey only a very inadequate idea of hepatic colic. Nowhere, furthermore, do we find mention of concretions in the bile passages and in the gallbladder. The discovery of stones in the stools is mentioned, and we are possibly justified in seeing a connection between the two. In view of the fact that the old Greek physicians were good clinical observers, and at the same time thorough students of anatomy, we are forced to the conclusion that gallstones were less frequent in those days than they are in modern times. An explanation for this difference might be sought in the mode of life. The people

1. Read at the meeting of the Roswell Park Medical Club, February 1, 1904.

of Greece ate other food, and their whole regimen was different from ours. The food, moreover, was prepared in an entirely different manner than nowadays, and meals were simpler. Physical exercise was an important factor during the years of adolescence, forming a large part of the curriculum of every youth, and in later years men continued to indulge in much and varied physical exertions. The clothing, too, did not cause anything that is analagous to our corset liver.

In the middle ages medicine made very little progress. It, therefore, does not astonish us to find little mention of gallstones. According to Donatus, it is true, Gentilis of Foligno, in the fourteenth century is said to have found stones for the first time in the gallbladder and the cystic duct of a corpse that he was reëmbalming. No definite statement in regard to the discovery is made in his works. In the fourteenth and fifteenth centuries, concretions were discovered in other organs. Gallstones were first mentioned by Antonine Benevenius, who died in 1592. He describes gallstones that were seen in the case of a woman, who had been a sufferer from pain in the abdomen. They were found both in the gallbladder and in a sacculated cavity on the surface of the liver. He assumes that the death of this patient was caused by these stones. Coelius Rodiginus and Lange subsequently reported similar cases. Vesalius and Fallopius, in spite of their exhaustive anatomic knowledge, say very little on concretions in the liver and the gallbladder.

Fernelius, in 1554, gave a very good description of gallstones and the symptoms which they produce. He seems to be familiar with the fact that occlusion of the ductus choledochus leads to a swelling of the gallbladder, a white discoloration of the feces, and the passage of dark urine, and that, in the occlusion of the hepatic duct, the gallbladder is empty. He further states that the concretions seen in the gallbladder are usually black and of a low specific gravity, so that they always float on water. This is a wrong impression. He thought that they formed from the bile when the fluid could not be evacuated, or remained too long in the gallbladder, or was not renewed. He also states that they are especially seen where an occlusion of the cystic duct exists. Columbus, who died in 1557, reports that in the liver, portal vein, kidneys, and lungs of the Jesuit general, Ignatius de Loyola, numerous concretions were found. Matteoli believed that the formation of stones occurred from the thickening of the bile and mucus as a result of an elevated bodily temperature. In this respect he followed the old theory of Galen, who assumes that all concretions of the body are formed in this manner. The same author makes the statement that stones in the gallbladder are probably more common than is usually assumed, and that they frequently pass unnoticed or are confounded with intestinal concretions.

Paracelus, freeing himself from the influence of Galen, introduced clinical observations and chemical investigations into the study of medical phenomena. He brought about a complete revolution of views in regard to the nature of gallstones. His doctrine of "tartarus" is based on the belief of chemical changes within the organism. He mentions (1563) stones in the liver as precipitates of impure material brought about in the same way as wine-stones from wine. Parcelsus was familiar with jaundice and the attack of colic that accompany gallstones. He believed in heredity as a predisposing cause of gallstones. He treated his patients by regulating gallic digestion by the administration of amara, acids, and carbonated waters.

Johann Kentmann, in 1665, describes gallstones of different sizes and shapes, and illustrates them. He states that the more numerous they are, the more they assume the angular shape. Ferrandus, in 1570, published a case of rupture of the gallbladder caused by a large gallstone, followed by pouring out of the bile into the peritoneal cavity. From this time on the nature, character and causation of gallstones was more definitely understood. Stones of various shapes and sizes were described by different authors. Branched stones were found in the hepatic duct, that were difficult to remove, so they remained in the body until the death of the animal or human being. The anatomical observations of Glisson (1654) were of fundamental importance in the further development of our knowledge of gallstones.

Ettmüler states that icterus does not necessarily follow gallstones, and that gallstones may be present in the gallbladder without causing icterus. He bases these statements on his knowledge of the fact that the gallbladder can be extirpated without endangering the life of the animal, and he quotes the important experiment of one of the students in Leyden, who extirpated the gallbladder in a dog without observing any bad results. According to this author, there is no remedy for gallstones. The discovery of glands in the walls of the gallbladder and the bile passages was important for the understanding of gallstone pathology. We are indebted to Morgagni for this discovery.

Sydenham gave us much light on the pathology of gallstones, but considered gallstone colic as a hysterical symptom. He describes the symptoms of cholelithiasis at great length, mentioning icterus, but says nothing whatever of gallstones. In the second half of the eighteenth century, Haller and Morgagni gave us important data on the pathology of the diseases of the gallbladder. They give a detailed description of the appearance, color, form, and structure of gallstones, and opposed the erroneous idea prevalent at that time, that dark stones are seen in old people and light ones in young subjects.

The surgical treatment of gallstones was inaugurated by Sharp and Moranel. Bloch, in 1774, proposed the artificial formation of adhesions in the region of the gallbladder, ligation of cystic duct, and the incision and extirpation of the gallbladder as early as 1767. About fifty years later Meckel von Heimebach emphasised for the first time the significance of catarrh in the formation of concretions. In recent times, Charcot led the French investigators in diseases of the gallbladder. However, Kocher and Sims are really the fathers of modern gallstone surgery, since they first attempted cholecystotomy. Recent surgeons have contributed much to our knowledge on the subject. The microörganisms most frequently found are members of the colon group and the bacillus typhosus. So constantly is one of these forms present that Gilbert and Fournier divided cholelithiasis into two groups; first, those due to the colon bacillus, and second, those due to the bacillus typhosus.

The two most common ways of bacterial infection of the gallbladder are by way of the bloodcurrent, or from the intestine. Since the bacillus coli is the microörganism that is most frequently seen in the gallbladder it would seem most probable that it entered from the intestine. Among the other most frequently mentioned causes of gallstones are stasis, constriction of the body by tight clothing, pregnancy, sedentary habits, certain diseases as gout, rheumatism, diabetes, obesity, arteriosclerosis, and advancing years.

### SYMPTOMS.

Symptoms appear when the stones are causing inflammation and ulceration of the walls of the bile passages, or when stasis of bile occurs, and, as a result, pus-forming microbes gain an entrance into the bile passages and develop there, causing violent inflammatory reaction. Gastric and intestinal disturbances are of uncertain value as symptoms,—there may be no sensation of heaviness in the hypochondriac region. If it appear, however, this sensation changes its location with the changes in the location of the body. The patient may complain of dull pain in the right portion of the epigastric region, radiating toward the hypogastrium, the thoracic organs, the right shoulder, or the lumbar region. Slight errors of diet may be followed by nausea and vomiting of bile-colored masses.

The attack of gallstone colic usually begins with violent pain. Suddenly, as a rule, after midnight, or during the late afternoon hours, a boring, stabbing, or tearing pain is felt, this usually being so violent that the patient becomes very excited, screams, shrieks, or moans. Pain is frequently localised in the right hypochondriac region in the sight of the gallbladder. In some cases it appears to be in the epigastrium, or it may even be felt in the left hypochondriac region, or in the mamma. It radiates in all directions, towards the abdomen, chest, back; occasionally toward the right shoulder, the extremities, and, less frequently, toward the genitalia.

The pain may remit temporarily only to be renewed with greater violence. It is chiefly due to the spasmodic contraction of the musculature of the bile passages, and to pressure on the sensitive mucous membrane. The demonstration of the presence of gallstones in the stools is, of course, of paramount importance in determining the cause of an attack of colic. They are not always found in gallstone colic.

### COMPLICATIONS.

Complications may consist in infection of the bile passages by virulent pathogenic bacteria, followed by cholecystitis and cholangitis, or by formation of abscess of the liver. If the stone becomes impacted, we may get ulcerations, strictures, formation of pockets and separation of parts of the ducts. Grave icterus may occur. Carcinoma, typhoid fever, cholera, or pneumonia may complicate. One of the most important complications is the formation of fistulous tracts between the gallbladder and the bile passages on the one hand, and the intestinal tract on the other. The most frequently formed fistulæ are between the gallbladder or the common duct and the duodenum. An abdominal fistula may form, through which stones may be passed. Cirrhosis of the liver is not infrequently seen. Impaction of the intestine may cause ileus.

### PROGNOSIS.

As cholelithiasis generally runs its course without disturbance and most rarely causes complicating inflammation of the bile passages, and the tissues surrounding them, the prognosis, as a rule, is favorable. Complications make the prognosis less favorable.

### DIAGNOSIS.

In the diagnosis of cholelithiasis all the symptoms must be considered. If the gallstones remain quiescent, the diagnosis may be very difficult. An attack of colic may be the first positive sign to reveal the presence of gallstones. The other symptoms come in their regular order.

### TREATMENT.

The treatment of gallstones will depend greatly upon the presence or absence of complications; that is, whether the object desired is simply to remove gallstones that may be present, and thus do away with the symptoms that are caused by their presence, or whether the disease is complicated by cholecystitis, and cholangitis, and it is desired to cure or relieve these conditions. In the former, the regular form of gallstone disease, dietetic measures, the drinking of certain waters, and treatment with drugs must be chiefly considered. In rare cases only will it be necessary to consider the advisability of surgical measures from the beginning. The most suitable diet seems to be a mixed one, containing plenty of proteids not too scanty or uniform, so that an abundant quantity of bile acid is produced, and so that the flow of bile may be stimulated and maintained. It is a good plan to advise frequent meals, only three hours apart, during the course of the day. Plenty of pure water should be ingested.

As gallstones are soluble in alkaline fluids, alkalies were recommended for the treatment of gallstones, even as early as the eighteenth century. They are employed mainly in the form of different mineral waters. Warm saline waters have also been recommended for this purpose. The administration of fats, particularly of certain oils, has been highly recommended. Their value is still uncertain. Turpentine and chloroform have their advocates. The duty of the physician is, first to alleviate the suffering, then to attempt to cut short the attack by promoting the passage of the stone and, finally, to prevent complications. For pain, morphine, belladonna, antipyrin, and phenacetin, and preparations of the salicylates are of value. Inhalation of chloroform should be administered only in cases of exceptional excitement. Heat and cold may give relief. The bowels should be freely opened. If these means do not afford relief, surgical interference should be advised. Operative procedures should be at once advised,—if signs of suppurative cholangitis, of cholecystitis, or if incipient peritonitis appear.

Dr. B. F. Curtis in a recent article (BUFFALO MED. JOUR., Febary, 1904), sums up the advantages of operation before grave infection has taken place in this manner: (1) the avoidance of such accidents and the improvement of existing inflammatory conditions; (2) the removal of the stone before it enters the common duct, with the dangerous consequences likely to follow; (3) the prevention of severe attacks of colic and cystitis; (4) stoppage of calculus formation; (5) the fact that latent cases are by no means free from danger, so that recovery from one attack of colic is no evidence that another one will not occur, or that other stones may not remain behind, or chronic cholecystitis persist; (6) it seems to be the general experience that in the latent cases while the patient does not have symptoms pointing directly to the bile passages, they are afflicted with various dyspeptic complaints which can be entirely and completely removed by operation.

I will conclude this paper by offering a report on a recent case:

Family history negative, except that of mother, who died at 36 years of age with stomach trouble, the exact nature of which is not known, probably, however, of gallstones. Personal history: woman, 38 years old, German, married, mother of four children, three living and healthy, one died in infancy of pneumonia. Patient came to United States when 25 years old. One year before coming here she had fever, jaundice, and pain in region of liver. Her side never felt quite well since that time. It felt cold and at times heavy. Pregnancy made no difference with it. Always worked hard and called herself healthy up to the present attack. Never had biliary colic.

Present attack: October 3, 1903, I was called to see this woman. Found her in bed suffering from pain, which was referred to stomach, vomiting and chills which were followed by fever. Upon examination I found a tumor which extended from the hypochondriac region into the left umbilical region. It was freely movable. After a few days rest in bed the tumor was much reduced in size and at times could not be found. At other times it could be felt to the right and above the umbilicus.

On October 17, Dr. Rooth operated and found the gallbladder as large as a man's fist, adherent to the liver and surrounding tissues, the walls ¾ inch thick. The mucous lining of the bladder was thickened and denuded, lying in loose folds in the bladder. I exhibit 115 stones that were removed. Three are the size of horse-chestnuts, the others smaller. This large stone lay in the bladder at the beginning of the duct and was so tightly wedged in that none of the small stones could get past it and into the duct to cause pain, hence no biliary colic occurred. There was room for bile to pass it, so there was no jaundice. The weight of the whole number of gallstones was 800 grains. The three largest had an average weight of 150 grains each. This woman made a good recovery and is now doing her own work, caring for a family of five.

544 Plymouth Avenue.

### DISCUSSION.

Dr. William Meisburger.—I remember a woman who presented no particular symptoms, and upon whom Dr. Mynter operated and found four large and two hundred small gallstones. After some time the wound closed, but a sinus formed later and bile discharged freely for some weeks, then closed again. She made ultimate recovery. I follow the rule of giving oil. I gave my last three patients half a pint at once. I had an old lady patient who had severe gallstone colic, but absolutely refused operation when it was proposed by Dr. Mansperger. I then ordered her to take one pint of olive oil at once, which she did. Next morning there was a large mass passed by the bowels, whether gallstones or not, I do not know. She had no more trouble for two years.

Dr. Thomas Bagley.—I had a patient in whom all the symptoms were referred to the stomach. She could not digest a bit of food. I took her to a specialist, but he was not positive. A second specialist applied the *x*-rays, but discovered no gallstones. Dr. Park then was asked to see her and he made a diagnosis of gallstones. He operated and found several good sized ones. The patient since then has been much better. The great majority of cases occur in women. Dr. Kehr, of Germany, gives the death rate of 6 per cent. Statistics, which I have been able to collect, give but one death in 126 cases.

Dr. James E. King.—There is a difference in the number of cases occurring in male and female as 15 women to 6 men. Gallstones are found with frequency at autopsies without diagnosis having been made during life. In the case of a patient of Dr. J. H. Potter, we operated for uterine fibroid, and finding also gallstones we removed the gallbladder. The proper treatment is surgical. The percentage of deaths is extremely low and is generally due to complications. In simple cases the prognosis is very good. In Dr. Daggett's own person there was severe pain for four months, which was only relieved by morphine. The tissue surrounding gallbladder was friable and the pancreas was somewhat hardened. Gallstones gave him very little trouble, but he was in a bad general condition, having suffered so long that he was hardly expected to recover, and yet his suffering demanded operation. A physician recently had a case at a hospital in which a diagnosis of floating kidney was made. Incision was made through peritoneum and a mass found within which were gallstones in the bladder. Many such cases prompted Dr. A. A. Jones to present his paper on mistakes in diagnosis.

Dr. J. H. Potter.—I have had two gallstone cases within the last five or six weeks. In the case Dr. King operated for me, there were no symptoms until gallstones were found by operation for fibroid. The other case had unusual features. The woman had an attack of colic a year and a half ago, a few months later a second attack, and another attack a month ago. Pain continued and a tumor developed which increased rapidly in twenty-four hours. Dr. Eugene A. Smith operated and found adhesions, making it difficult to discover the gallbladder. This was due to the inflammatory condition. One stone was found which blocked up the cystic duct. This was opened and drained. The patient recovered. When one is positive of diagnosis, surgical intervention is advisable.

Dr. T. H. McKee.—Gallstone conditions are very similar to those obtaining in appendicitis, and the indications for early operation when the diagnosis can be established are the same; early operations being comparatively safe, while later ones are notoriously uncertain. Personally, I am very seldom called upon to treat a stomach *per se,* almost invariably finding trouble in the appendix, gallbladder or some other organ, when the patient refers his distress to the stomach. I believe it to be one of the most tolerant and long suffering organs in the body and needs about the least attention.

I have succeeded in spoiling two or three cases from an operator's point of view by giving large and frequent doses of olive oil. Symptoms were relieved and they have not recurred. At present the patron of this club is doing more than any one else to popularise the radical operation for gallstones, that is, total extirpation of the gallbladder. The procedure is entirely rational and yields results that are all that could be desired in appropriate cases.

Dr. James A. Gibson.—A very interesting part of Dr. Dignen's paper was the account of the case he reported. One rarely finds a typical case. Primary disease of the stomach is not, I believe, so frequently met with as is thought. Symptoms referred to the stomach are often due to appendicitis. The treatment by olive oil often works well. A physician in this city always takes a swallow of it after each meal to prevent a recurring attack, having had but one so far. Pain is generally developed within the thickened wall of the gallbladder. It may be referred to the gallbladder or elsewhere.

Dr. Dignen (closing the discussion). With regard to olive oil, its use is of doubtful benefit. I have had half a dozen patients in my short experience in medicine and have tried all methods of treatment. Most benefit was attained in my hands from the saline treatment. I had one patient weighing 200 pounds. Stones were found in washing the stools. She had taken quantities of olive oil, nevertheless she had colic once or twice a week. She dreads operation. Oil did her no good at all. I never gave her as much as a pint at a time, but three or four ounces a day. The effect of oil is uncertain. It is doubtful whether it ever gets into the gallbladder. Its acts by increasing peristalsis in the gallbladder wall itself. The really beneficial treatment, once diagnosis is established, is surgical intervention.

---

ANTIRHEUMATIC PRESCRIPTION.

Kali iodid .................................................. ½ oz.

Kali acetat .................................................. 1 oz.

Tongaline, q. s. ad.......................................... 6 ozs.

M. Sig.—A teaspoonful three times a day.

